# Lack of Genotoxic and Carcinogenic Potential for Nonsugar Sweeteners: A Review of Animal and Mechanistic Evidence

**DOI:** 10.1016/j.advnut.2025.100552

**Published:** 2025-11-04

**Authors:** Satori A Marchitti, Denali Boon, Maia Jack, Julie E Goodman

**Affiliations:** 1 Gradient, Boston, MA, United States; 2 American Beverage Association, Washington, DC, United States

**Keywords:** artificial sugars, nonsugar sweetener (NSS), low- and no-calorie sweetener (LNCS), cancer, genotoxicity, carcinogenicity, toxicology, animal cancer bioassays, key characteristics of carcinogens

## Abstract

Globally, nonsugar sweeteners (NSSs) are commonly used in foods and beverages to enhance sweetness without added calories. NSSs have been the subject of numerous in vitro and animal studies to assess their potential carcinogenic risk to humans. As a complement to a larger systematic evaluation of the epidemiology evidence of NSSs and cancer, here we present a comprehensive review of the available experimental evidence from animal and mechanistic studies for the NSSs acesulfame-K (Ace-K), advantame, aspartame, cyclamate, neotame, saccharin, steviol glycosides, and sucralose within the context of ingredient safety. For this evaluation, we focused on genotoxicity, other potential cancer modes of action (MoAs), and carcinogenicity. Overall, high-quality studies have not shown evidence for carcinogenicity in animal models, except for saccharin, which causes bladder tumors in rats via a mechanism not relevant to humans. There is also no consistent or compelling evidence for any biologically plausible MoA by which any of these NSSs could cause cancer in humans. The results of this evaluation are consistent with the results of epidemiology studies, which have shown no consistent associations between NSS intake and cancer risk. Taken together, the body of available evidence supports previous conclusions by authoritative and regulatory bodies that Ace-K, advantame, aspartame, cyclamate, neotame, saccharin, steviol glycosides, and sucralose do not pose a genotoxic or carcinogenic risk to humans.

**Statements of Significance**

We provide a review and synthesis of the current status of animal and mechanistic evidence together for 8 common NSSs and cancer, and provide insights into recent controversies and developments in the field; this evaluation contributes to the overall body of evidence related to nonsugar sweeteners and cancer.

## Introduction

There are currently 6 artificial nonsugar sweeteners (NSSs) authorized as direct food additives by the United States Food and Drug Administration (FDA) that are considered safe for the general population under the listed conditions of use [1]. These include advantame, aspartame, acesulfame-K (Ace-K), neotame, saccharin, and sucralose (Table 1). Although not currently approved in the United States, cyclamates are considered safe for use elsewhere, including the European Union (EU). United States FDA also recognizes several types of plant and fruit-based high-intensity sweeteners (e.g., some steviol glycosides obtained from the leaves of the Stevia plant) as generally recognized as safe (GRAS) through its GRAS notification program [Under sections 201(s) and 409 of the Federal Food, Drug, and Cosmetic Act, any substance that is intentionally added to food is considered a food additive and is subject to premarket review and approval by US FDA, unless the substance is generally recognized by qualified experts as having been adequately shown to be safe under the conditions of its intended use (i.e., GRAS) [1]. The use of a food additive may be GRAS either through scientific procedures or, for substances used in food before 1958, through its historical and common use in food].

**TABLE 1:** Uses, designations, and acceptable daily intakes of nonsugar sweeteners in the United States and EU

NSS	United States FDA approval	Designation	Uses	ADI (mg/kg/d)	Packets at max ADI^1^	Source
Ace-K	1988	Food additive	General-purpose sweetener and flavor enhancer, not including meat/poultry; heat stable	0–15 (United States FDA) 0–15 (JECFA) 0–9 (SCF)	23	[[1], [2], [3]]
Advantame	2014	Food additive	General-purpose sweetener and flavor enhancer, not including meat/poultry; heat stable	0–32.8 (United States FDA)	4920	[1]
Aspartame	1974	Food additive	General-purpose sweetener	0–50 (United States FDA) 0–40 (JECFA) 0–40 (EFSA)	75	[1,4,5,6]
Cyclamate	Not approved in the United States^2^	Food additive	Foods, beverages, tabletop use	0–11 (JECFA) 0–7 (SCF)	–	[7,8]
Neotame	2002	Food additive	General-purpose sweetener and flavor enhancer, not including meat/poultry; heat stable	0–0.3 (United States FDA) 0–10 (EFSA)	767	[1,9]
Saccharin	1977^3^	Food additive	Beverages, sugar substitute for cooking, tabletop use, processed foods	0–15 (United States FDA) 0–5 (JECFA) 0–9 (EFSA)	45	[1,10,11]
Steviol glycosides	GRAS^4^	GRAS (United States); food additive (EU)	General-purpose sweeteners	0–12 (United States FDA)^5^ 0–4 (JECFA)^5^ 0–4 (EFSA)^5^	27	[1,12,13]
Sucralose	1998	Food additive	General-purpose sweetener; heat stable	0–5 (United States FDA) 0–15 (SCF)	69	[1,14,15]

Abbreviations: Ace-K, acesulfame-K; ADI, acceptable daily intake; EFSA, European Food Safety Authority; EU, European Union; GRAS, generally recognized as safe; JECFA, Joint FAO of the United Nations (FAO)/WHO Expert Committee on Food Additives; NSS, nonsugar sweetener; SCF, Scientific Committee on Food; US FDA, United States Food and Drug Administration.

1 Number of sweetener packets a 60 kg (132 lb) person would need to consume to reach the max ADI established [1].

2 Cyclamates are not approved in the United States; they were first evaluated by JECFA in 1978, when a temporary ADI was established.

3 Saccharin was first discovered in 1879 and became widely used; the United States FDA began to regulate saccharin in 1977.

4 United States FDA considers steviol glycosides as GRAS; they were reviewed by JECFA in 2000, when an ADI was established.

5 Expressed as steviol equivalents.

Overall, artificial NSSs are considered some of the most widely studied and tightly regulated food additives in the global human food supply, with United States FDA and other authoritative bodies [e.g., Joint FAO of the United Nations/WHO Expert Committee on Food Additives (JECFA), European Food Safety Authority (EFSA)] having evaluated hundreds of studies designed to identify a wide range of possible toxic effects, including those related to genotoxicity and carcinogenicity. Unlike the GRAS notified NSSs, artificial NSSs must, by law, undergo an extensive premarket approval process before they are deemed safe for consumption [1]. This process involves extensive safety and toxicity testing. In addition, United States FDA is tasked with continually monitoring the scientific literature for new information and re-evaluates the state of the science related to each sweetener when warranted.

For each sweetener, United States FDA and other authoritative bodies (e.g., JECFA, EFSA) have established an acceptable daily intake (ADI) (Table 1), which is the amount of a substance considered safe to consume on a daily basis over a lifetime without adverse effects. That is not to say that exceedance of an ADI will result in adverse effects, but rather that there is an adequate margin of safety factored into the ADIs. ADIs are typically derived by analyzing animal toxicity studies to determine the highest exposure levels shown to have no adverse effects in a particular animal species, with additional safety factors applied to account for differences between animals and humans and for varying sensitivity among humans. As part of this process, agencies may rely on data from different animal species or use slightly different safety factors, resulting in varying ADIs. For sweeteners approved as food additives, authoritative bodies have concluded that human consumption of these NSSs does not exceed established ADIs, even at the highest consumption levels.

The potential genotoxicity and carcinogenicity of all NSSs have been investigated extensively across numerous studies, with new data and authoritative assessments updated regularly. Recently, we conducted a systematic review of the available epidemiology evidence on NSSs and all cancer types [16]. In the present review, we conducted a high-level evaluation of the available experimental evidence from animal and mechanistic studies for the NSSs Ace-K, advantame, aspartame, cyclamate, neotame, saccharin, steviol glycosides, and sucralose, to determine whether it is biologically plausible that any of these NSSs could cause cancer in humans. For this evaluation, we focused on genotoxicity, other potential cancer modes of action (MoAs), and carcinogenicity.

## Methods

As a complement to a larger systematic review of the epidemiology evidence of NSSs and cancer [16], we conducted a high-level evaluation of available carcinogenicity and mechanistic (e.g., genotoxicity) evidence from animal and in vitro studies to determine whether the experimental evidence supports the biological plausibility of an association between any NSS and cancer in humans. The evidence was reviewed separately for each of the following NSSs: Ace-K, advantame, aspartame, cyclamate, neotame, saccharin, steviol glycosides, and sucralose. For this evaluation, we reviewed government and other health and authoritative agency [e.g., United States FDA, International Agency for Research on Cancer (IARC)] documents, along with peer-reviewed weight-of-evidence analyses, systematic reviews, and primary studies.

Regulatory and authoritative body (e.g., United States FDA, EFSA, JECFA, IARC) assessments and other documents were identified for each NSS from their respective agency databases. Relevant systematic reviews and weight-of-evidence analyses published in English related to the potential carcinogenicity of the NSSs (published either before or after the latest authoritative conclusion) that evaluated either animal and/or mechanistic data, were identified using PubMed and Scopus databases with the search terms "acesulfame-K," "advantame," "aspartame," "cyclamate," "neotame," "saccharin," "steviol glycosides," and "sucralose," in combinations with the keywords "animal," "mechanistic," "genotoxicity," "carcinogenicity," "cancer," and "key characteristics of carcinogens." [The key characteristics (KCs) of carcinogens paradigm is based on the concept that human carcinogens often demonstrate at least one of 10 characteristics: is electrophilic or can be metabolically activated to an electrophile (KC #1), is genotoxic (KC #2), alters DNA repair or causes genomic instability (KC #3), induces epigenetic alterations (KC #4), induces oxidative stress (KC #5), induces chronic inflammation (KC #6), causes immunosuppression (KC #7), modulates receptor-mediated effects (KC #8), causes immortalization (KC #9), or alters cell proliferation, cell death, or nutrient supply (KC #10).] Similar independent literature searches were used to identify primary animal and mechanistic studies published since the latest authoritative conclusion and/or systematic review. Studies were included for evaluation if they presented (or evaluated) NSS evidence from animal carcinogenicity studies (i.e., animal cancer bioassays) or from mechanistic studies relevant to carcinogenesis (with a primary focus on genotoxicity). Study quality was not used to exclude studies or documents from evaluation; rather, considerations for data quality, reliability, and relevance are discussed, with a focus primarily on authoritative and weight-of-evidence conclusions. We included studies and documents published through September 2025. The reference lists of these documents were also used to identify additional studies. As part of this process, most of the experimental data considered were identified via secondary sources (i.e., authoritative agency documents and review articles).

The overall weight of evidence for each NSS was evaluated to: *1*) determine the genotoxic and carcinogenic potential of the NSS; and *2*) determine whether it was biologically plausible that the NSS could cause cancer in humans. In this process, we first evaluated the data relied upon in the identified regulatory and authoritative agency documents, along with the overall agency conclusions for each NSS concerning genotoxicity and carcinogenicity, with a particular focus on discussions related to data quality, reliability, and relevance. Second, we evaluated the data and conclusions presented in the identified systematic reviews and weight-of-evidence analyses related to NSSs and cancer. Recent studies published after the latest authoritative assessment and/or systematic review were similarly evaluated within the same context. This information was then integrated and synthesized together to form a weight-of-evidence conclusion for each NSS regarding its genotoxic and carcinogenic potential and the overall biological plausibility it could cause cancer in humans. The protocol for the overall evaluation of the epidemiology [16] and experimental evidence (herein) for NSSs and cancer was registered with Open Science Framework (OSF) on 29 September, 2023 (https://osf.io/gc8v6).

## Results

Below, we provide the results of our evaluation of the animal and in vitro experimental and mechanistic evidence related to carcinogenesis for the NSSs Ace-K, advantame, aspartame, cyclamate, neotame, saccharin, steviol glycosides, and sucralose.

### Acesulfame-K

#### Regulatory status and ADI

Ace-K was first approved by United States FDA in 1988 for use as a tabletop sweetener, ingredient in dry bases for beverages, and ingredient in various foods (exception confections), and was later approved in 2003 as a general-purpose sweetener and flavor enhancer broadly in beverages and food, not including meat and poultry [1,17]. In the approval process, United States FDA reviewed >90 studies that evaluated its safety, including those described in a 1991 JECFA report, a 2000 Scientific Committee on Food (SCF) report, and a 2025 EFSA report [2,3,18]. [The SCF was established in 1974 and was one of the independent committees providing the European Commission with scientific advice on food safety. In 2003, the responsibilities of the SCF were transferred to EFSA]. United States FDA [17,19] and SCF [3] concluded that Ace-K is not associated with any genotoxic or carcinogenic effects, and established ADIs of 0–15 and 0–9 mg/kg/d, respectively (Table 1). Since then, additional experimental studies, systematic reviews, and high-throughput screening (HTS) data continue to support this conclusion [18,[20], [21], [22], [23]].

#### Genotoxicity and mechanistic data

In vitro genotoxicity tests, including those evaluating mutagenicity, DNA damage, and DNA repair, have been negative for Ace-K [[24], [25], [26], [27]]. In vivo genotoxicity tests have also largely been negative, with SCF concluding in 2000 that an isolated positive mutagenicity finding in mouse bone marrow cells at a high dose was associated with study design flaws, and not reproducible in a subsequent follow-up study [3,20,21]. Since the 2000 SCF report, 3 in vivo micronucleus studies have been conducted in mice, including a 40-wk National Toxicology Program (NTP) study conducted in a transgenic mouse strain engineered to be susceptible to tumors [28]. These studies were negative, with the exception of a sex-specific (male mice only) increase in micronucleated erythrocytes; however, this effect has not been replicated in either sex in other mice, including tumor-susceptible mouse strains [20,21,24]. Subsequently, the NTP report concluded that the positive micronucleus response was "of uncertain biological significance" due to the weak nature of the increase and the lack of consistency across sexes [28].

Although other studies published after the 2000 SCF report have reported some positive results from in vivo genotoxicity tests examining DNA damage, these studies did not conform to Organization for Economic Cooperation and Development (OECD) recommended protocols and guidelines and are considered to have limited reliability [[20], [21], [22]]. In ToxCast/Tox21 HTS assays developed to evaluate the genotoxic potential of small molecules, Ace-K has been shown to be inactive in all 17 assays [21,23]. In a 2020 systematic review integrating available human, animal, and mechanistic data for Ace-K using numerous models and over 800 endpoints related to the 10 KCs of carcinogens paradigm, it was concluded overall that Ace-K lacked KC-related activity, including for genotoxicity [22]. [In Chappell et al. [22], data from each study or assay were extracted by model and endpoint, with a model defined as the experimental system used to evaluate a potential response from Ace-K exposure (e.g., Fischer rats, human A549 cells) and an endpoint defined as the specific output of an individual assay or measurement (e.g., activation/inactivation of a particular receptor, mutation frequency, enzyme activity/inactivity). One or more models may have been used in each study and each model may be assessed for multiple endpoints, with each endpoint relevant to one or more KCs.]. In a recent EFSA re-evaluation of Ace-K, which included a systematic evaluation of genotoxicity studies published since the SCF conclusion in 2000, EFSA concluded that Ace-K does not pose a concern for genotoxicity [18].

#### Animal carcinogenicity

The body of evidence supporting the lack of Ace-K genotoxicity is consistent with the results of standard 2-y animal cancer bioassays showing a lack of tumor response [22]. A total of 5 animal cancer bioassays examining the carcinogenic potential of Ace-K have been conducted in various animal species, including rodents and dogs, with all studies demonstrating a lack of carcinogenicity after chronic, high-dose exposure to Ace-K [[28], [29], [30], [31]]. These are reliable studies, having been performed with appropriate controls and measures [22]. In addition, NTP bioassays conducted in transgenic mice engineered to be particularly susceptible to carcinogen-induced tumors were negative [28].

#### Weight-of-evidence conclusion

When considering the available data on genotoxicity and the negative findings from animal carcinogenicity studies, the overall weight of evidence supports the conclusion that Ace-K lacks genotoxic and carcinogenic potential.

### Aspartame

#### Regulatory status and ADI

Since its first approval as a sweetener in 1974 by United States FDA (which was stayed until 1981, with approvals for broader applications including beverages to follow in 1983), numerous in vitro and animal studies have evaluated the genotoxicity profile and potential carcinogenicity of aspartame, and the available data have been extensively evaluated by a number of regulatory agencies, including United States FDA (ADI of 0–50 mg/kg/d), JECFA (ADI of 0–40 mg/kg/d), and EFSA (ADI of 0–40 mg/kg/d), all of which concluded that aspartame lacks genotoxic and carcinogenic potential [1,5,6,32,33] (Table 1). Recent systematic reviews and further studies have provided additional analyses that support this conclusion [20,21,[34], [35], [36], [37], [38]].

#### Genotoxicity and mechanistic data

In a weight-of-evidence systematic review integrating the available mechanistic data across hundreds of endpoints related to the 10 KCs of carcinogens, the overall evidence demonstrated that aspartame lacks KC-related activity, including for genotoxicity [38]. Although aspartame showed some activity for oxidative stress (KC #5) from mammalian data [36,38], these data were inconsistent, and data from human models showed no activity. In addition, oxidative stress is not unique to cancer, as noted by the original authors who developed the KC paradigm [39]. In general, genotoxicity tests have largely been negative for aspartame, including those evaluating mutagenicity, micronucleus formation, and chromosomal aberrations [21]. Although isolated positive results have been reported in some genotoxicity studies examining the same endpoints, critical study limitations have indicated they are not reliable for informing conclusions, with EFSA considering the responses to be due to nonspecific cytotoxic effects from the very high study doses used or the result of nonstandard methods that did not conform to OECD guidelines [6].

#### Animal carcinogenicity

The conclusion that aspartame lacks genotoxic potential and mechanistic activity related to KCs is consistent with the lack of carcinogenicity observed in standard animal cancer bioassays and other studies evaluating tumorigenic effects [20,37,38]. Of 10 rodent cancer bioassays that have evaluated aspartame, 7 Good Laboratory Practice- (GLP)-compliant studies conducted over bioassay periods of ≤2 y, including 3 by NTP in different strains of mice [40], 1 in Wistar rats [41], and 3 conducted at Searle Laboratories (as described in [6]), reported no increased incidences of tumors of any kind after chronic and high-dose exposure. In addition, 2 studies examining the potential promotion of bladder and pancreatic tumors by aspartame in rodent cancer models reported no effects related to aspartame [42, 43].

Three rodent cancer bioassay studies conducted by the Ramazzini Institute, using a nonstandard lifetime exposure study design have evaluated aspartame. Standard rodent cancer bioassays are 2 y in length. In lifetime exposure studies, rodents are dosed with the compound over the course of their lifetime until death, at which point organs and tissues are harvested. However, rodents are known to have increased pathological findings associated with aging, which can affect the interpretation of study results [44,45]. [In addition, limiting the harvesting and preservation of tissues until after natural death presents logistical issues. It has been estimated that many tissues are not processed for some hours (or more) after death, resulting in varying levels of tissue degradation that may impact the quality of pathological examinations [45].] The three lifetime exposure studies of aspartame reported increased incidences of a wide variety of tumors that the authors claimed were associated with aspartame exposure, including those of the liver, lung, renal pelvis, mammary gland, peripheral nerves, and hematopoietic system [[46], [47], [48]]. However, these studies have been heavily criticized and found to lack validity by regulatory agencies and authoritative bodies, as well as the overall research community, because of critical flaws related to the lifetime study design, methodology, conduct, and reporting [5,6,20,37,44,45,[49], [50], [51], [52], [53], [54], [55]].

In the Ramazzini Institute aspartame studies, the test animals developed spontaneous tumors and chronic inflammation at high background rates, and serious concerns have been raised over the accuracy of the pathology diagnoses and histological interpretations, the inappropriate aggregation of tumor incidences, the use of unvalidated statistical analyses, and the impact of various common rodent infections, such as *mycoplasma pulmonis*, which can mimic and/or cause tumors in laboratory animals [44,45,51,54,56].

In a recent in-depth analysis of these issues, it was concluded that the "RI [Ramazzini Institute] bioassay data, analyses, and interpretation provide neither compelling nor conclusive evidence that aspartame represents a carcinogenic hazard in rodents," further stating that "[b]y extension, [aspartame] is unlikely to pose a carcinogenic risk to humans" [44,54]. Furthermore, in a recent independent histological re-examination of tissues collected in a previous standard cancer bioassay [41], no tumorigenic effects were observed, including in organs reported by the Ramazzini Institute to be affected by aspartame [57].

Both the IARC Monographs Programme and JECFA evaluated aspartame in the summer of 2023, with a summary of their independent findings released on 14 July, 2023 [4]. The IARC Monographs working group classified aspartame as *possibly carcinogenic to humans* (Group 2B) and concluded that the evidence for cancer in experimental animals was *limited* [4]. However, JECFA noted its disagreement with IARC’s conclusions, stating that "the [JECFA] Committee concluded that there was no convincing evidence from experimental animal or human data that aspartame has adverse effects after ingestion," with the panel further stating that all the studies apart from those by the Ramazzini Institute showed negative results, and that the Ramazzini Institute studies all had limitations in their study design, execution, reporting, and interpretation [4]. Despite basing its conclusion of *limited* evidence in experimental animals solely on the 3 Ramazzini Institute studies, the IARC working group acknowledged the same concerns. United States FDA also disagreed with IARC’s conclusions, noting that United States FDA scientists reviewed the same data and identified significant shortcomings in the studies relied upon by IARC [1].

#### Weight-of-evidence conclusion

Systematic reviews integrating data from animal, mechanistic, and human streams of evidence continue to support a lack of carcinogenicity associated with aspartame [36,38] and, overall, the weight of evidence supports the conclusions previously reached by United States FDA, EFSA, and JECFA that aspartame lacks both genotoxic and carcinogenic potential.

### Cyclamate

#### Regulatory status and ADI

As a sweetener, cyclamate is used as its sodium or calcium salt, with sodium cyclamate predominantly used in foods and beverages. Globally, cyclamate is currently approved for use as a food additive in the EU, the United Kingdom, and over 100 additional countries, with JECFA (ADI of 0–11 mg/kg/d) and SCF (ADI of 0–7 mg/kg/d) confirming its safety [7,8] (Table 1). In the United States, cyclamate was originally considered as GRAS by United States FDA in the 1950s; however, this designation was removed in 1969 based on the interpretation of a controversial animal study (now considered flawed by the scientific community) that reported an increased incidence of bladder tumors in rats fed high doses of a 10:1 cyclamate-saccharin mixture [58,59]; a total ban on the use of cyclamate in all food and drug products by United States FDA occurred in 1970 [60] and was subsequently upheld in 1980 [61]. Several other countries banned cyclamate around the same time, including the United Kingdom and the Philippines, although the majority of these bans were lifted after the data from the Price et al. [58] study and other studies investigating the potential carcinogenicity of cyclamate were re-evaluated by the respective regulatory bodies [[62], [63], [64], [65], [66], [67], [68]].

#### Genotoxicity and mechanistic data

A number of agencies have reviewed the available genotoxicity data for cyclamate, including JECFA, SCF, IARC [7,8,62], and, more recently, Food Standards Australia New Zealand (FSANZ) [66] and EFSA [69]. In its 1999 evaluation, IARC stated that, although some in vitro genotoxicity tests were positive in mammalian cells, cyclamate did not show evidence of genotoxicity in rodents in vivo or in human peripheral lymphocytes isolated from volunteers [62]. Since then, the positive results reported for some in vitro genotoxicity tests, including those conducted after the 1999 IARC evaluation, have been considered of limited relevance and reliability, primarily due to study design and methodological issues, including the use of methods not validated for regulatory use and tests now considered obsolete (e.g., the in vitro unscheduled DNA synthesis test) [20,69], with the overall conclusion that cyclamates are not mutagenic or clastogenic (i.e., not genotoxic) [59,66,70], with recent systematic reviews and weight-of-evidence analyses continuing to support this conclusion [20].

#### Animal carcinogenicity

In 1999, IARC concluded that there was inadequate evidence in experimental animals for the carcinogenicity of cyclamate, as no treatment-related increases in tumors were observed in a number of animal cancer bioassays considered of sufficient quality for evaluation, including multiple multigeneration studies designed specifically to evaluate the production of bladder and other tumors [62]. Additional international bodies have evaluated the available animal evidence for the carcinogenicity of cyclamate, including the United States FDA Cancer Assessment Committee of the Center for Food Safety and Applied Nutrition, the Food Additives and Contaminant Committee of Great Britain, the National Academy of Sciences-National Research Council, and FSANZ [[63], [64], [65], [66]]. These comprehensive evaluations have consistently concluded that the overall totality of the evidence indicates that cyclamate is not carcinogenic in any organ or tissue in either sex of any animal species tested. Although the Price et al. [58] study, which formed the basis of the 1970 United States ban, reported increased bladder tumors in rodents fed a high-dose cyclamate-saccharin mixture, the interpretation of these results was subsequently considered unreliable due to various critical issues related to study design and the health of the animals, including the presence of urinary tract calcification and bladder parasites in the animals with tumors, both conditions known to contribute to the development of bladder tumors in rats [59]. In addition, subsequent replicate bioassays conducted around the world that were well designed and well controlled could not reproduce the results of the Price et al. [58] study, with agencies concluding overall that these mixtures are not carcinogenic in rats and no further bioassays of this kind were necessary [64,65]. Additional rodent cancer bioassays investigating cyclamate alone have also not shown increased bladder or other tumors [67], and no evidence of carcinogenesis has been observed in long-term feeding studies of cyclamate in other species, including nonhuman primates and dogs [59,68]. Taken together, it was concluded that strong evidence exists that cyclamate is not carcinogenic in animals [59,66].

#### Weight-of-evidence conclusion

The available data on genotoxicity and the overall findings from animal carcinogenicity studies support the conclusion that cyclamate lacks genotoxic and carcinogenic potential.

### Neotame and advantame

#### Regulatory status and ADI

Neotame and advantame were developed as *N*-substituted derivatives of aspartame and were approved by United States FDA in 2002 and 2014, respectively [71,72]. Evaluations of the genotoxic and carcinogenic potential of neotame and advantame have been conducted by United States FDA, JECFA, and EFSA, concluding that neither compound poses a concern for genotoxicity or carcinogenicity [[73], [74], [75], [76]]. The ADI for advantame is 0–32.8 mg/kg/d (United States FDA); the ADIs for neotame are 0–0.3 (United States FDA) and 0–10 (EFSA) mg/kg/d (Table 1).

#### Genotoxicity and mechanistic data

In vitro and in vivo genotoxicity tests of advantame and neotame, and of their metabolites and degradation products, following GLP, OECD, and United States FDA test guidelines, have all been negative, demonstrating no evidence of genotoxicity, such as mutagenic activity or increased micronucleus formation in bone marrow cells [20,73,74,77]. Studies evaluating the potential formation of *N*-nitrosamine compounds in the gastrointestinal tract from the reaction of nitrite with neotame, advantame, or their metabolites/degradation products, have indicated a low potential for these compounds to be formed under the biological conditions present in the human stomach, with further genotoxicity testing of the potential nitrosated compounds demonstrating no evidence of genotoxicity [73,74]. EFSA concluded from these studies that if any nitrosation of neotame and advantame were to occur, it would not be of concern. In a 2025 re-evaluation of neotame, EFSA concluded that there is no concern for genotoxicity, with no adverse effects for other endpoints noted at the highest doses tested, subsequently raising the ADI from 2 to 10 mg/kg/d [9].

#### Animal carcinogenicity

Results from rodent carcinogenicity studies of neotame and advantame are consistent with the lack of genotoxicity findings [78]. Chronic exposure to neotame and advantame at high doses in rat and mouse 2-year cancer bioassays has not caused treatment-related increases in the incidences of tumors, supporting the conclusion that neotame and advantame lack carcinogenic potential [20,[71], [72], [73], [74]].

#### Weight-of-evidence conclusion

When considering the available data on genotoxicity and the negative findings from animal carcinogenicity studies, the overall weight of evidence supports the conclusion that neotame and advantame lack genotoxic and carcinogenic potential.

### Saccharin

#### Regulatory status and ADI

Saccharin was first discovered in 1879 and became widely used as a sweetener, with United States FDA (ADI of 0–15 mg/kg/d) regulation beginning in 1977 (Table 1). Several agencies have evaluated the genotoxic and carcinogenic potential of saccharin and its salt forms (i.e., sodium saccharin and calcium saccharin) from available in vitro and animal studies, including JECFA in 1993 (ADI of 0–5 mg/kg/d), SCF in 1995 (ADI of 0–5 mg/kg/d), IARC in 1999, and NTP in 2001 [10,62,79,80]. Most recently, saccharin underwent a re-evaluation by EFSA (ADI of 0−9 mg/kg/d) [11]. SCF, JECFA, and EFSA concluded that sodium saccharin does not raise a concern for genotoxicity [10,11,79], and NTP concluded that saccharin is not anticipated to be a human carcinogen [80]. Several recent systematic reviews and additional studies also support the overall conclusions by SCF and JECFA that saccharin is not genotoxic [20,21,23].

#### Genotoxicity and mechanistic data

The conclusion that saccharin lacks genotoxic potential is supported by generally negative results from in vitro and in vivo genotoxicity tests, including those designed to detect DNA damage, mutagenicity, and clastogenicity [26,62,[81], [82], [83]]. Although some initial in vitro studies examining the genotoxicity potential of saccharin have reported positive results, particularly for chromosomal aberrations and sister chromatid exchanges, these results were subsequently considered by SCF to be due to changes in the osmolality of the in vitro system, resulting in nonspecific effects from the very high study doses used [79]. In addition, OECD recently removed the sister chromatid exchange test from its list of recommended genotoxicity tests due to uncertainty regarding the effect detected by the test [84]. Other studies reporting positive results have been noted to suffer from critical limitations and methodological flaws known to confound the results of genotoxicity assays, including decreased cell viability and the presence of contaminants in test compound solutions [27,62,79,85,86]. Inconsistent results have been reported for in vivo comet assays, with 1 study reporting the presence of DNA fragmentation in 2 (among 8) organs studied in mice [87] and another reporting negative results in rats at the same doses [83]. In an HTS study designed to detect genotoxicity induced by small molecules, saccharin was shown to be inactive in all 17 genotoxicity assays of the NTP ToxCast/Tox21 program [23]. In addition, sodium saccharin has been identified as a reference chemical for negative in vitro mammalian cell genotoxicity tests by the European Reference Laboratory for Alternatives to Animal Testing [88].

#### Animal carcinogenicity

Animal studies evaluating the carcinogenicity of saccharin have indicated that saccharin (acid form) and calcium saccharin do not increase the incidence of tumors and are not carcinogenic [20,62]. IARC’s current overall classification of saccharin and its salts as Group 3 (*not classifiable as to its carcinogenicity to humans*) is based on the agency’s 1999 re-evaluation in which the agency concluded that sodium saccharin produces species-specific urothelial bladder tumors only in rats via a non-DNA-reactive mechanism not relevant to humans, thus warranting a downgrade from its original classification as Group 2B (*possibly carcinogenic to humans*) [62]. Data from numerous animal studies indicate that sodium saccharin is a relatively weak carcinogen, producing small (<10%) and inconsistent increases in urinary bladder tumor incidences in rats only that are chronically exposed to doses ≥1%–3% in the diet, and only when exposure includes the neonatal period (i.e., ≤30 d of age) [20,62,89,90]. In addition, the rat urinary bladder is the only organ affected, that is, sodium saccharin does not produce tumors at any other site in experimental animals [62].

The mechanism of sodium saccharin-induced tumors in the rat bladder has been proposed to involve: *1*) the binding of saccharin to urinary proteins, resulting in, *2*) the formation of a urinary calcium phosphate-containing precipitate and associated crystals, which are abrasive to the bladder epithelium, leading to, *3*) cytotoxicity and resultant mild regenerative hyperplasia (i.e., increased cellular proliferation), ultimately resulting in, *4*) the production of bladder tumors in a small percentage of rats [62,91,92]. Specific rat urine characteristics appear to be critical for the formation of the precipitate, which initiates the sequence of tumor-inducing events, including a urinary pH ≥6.5, high urinary concentrations of calcium phosphate and protein, and high urinary osmolality. Because of critical interspecies differences in urine characteristics, this mechanism of carcinogenicity is not considered relevant to humans [62,93]. On the basis of this information, in 2000, NTP and the United States Department of Health and Human Services removed saccharin from the list of substances reasonably anticipated to be human carcinogens [80] and, in 2003, the California Environmental Protection Agency removed sodium saccharin from its Proposition 65 list of chemicals known to the state to cause cancer [89]. In its 2024 re-evaluation of saccharin, EFSA, noting the nonhuman-relevant mechanism of bladder cancer in rats and additional data, concluded that saccharin exposure is not likely associated with cancer in humans, subsequently raising the ADI from 5 to 9 mg/kg/d [11].

#### Weight-of-evidence conclusion

When considering the available data on genotoxicity and the overall findings from animal carcinogenicity studies, the weight of evidence indicates that saccharin is not genotoxic and lacks carcinogenic potential as it relates to humans.

### Steviol glycosides

#### Regulatory status and ADI

Steviol glycosides are compounds that naturally occur in the leaves of the *Stevia rebaudiana* (Bertoni) Bertoni plant, commonly known as Stevia. In the United States, over 50 high-purity (95% minimum purity) steviol glycosides, including Rebaudioside A, Stevioside, and Rebaudioside D, isolated from the leaves of the Stevia plant, are considered GRAS by United States FDA and are considered safe for use as general-purpose sweeteners [1,94,95]. These compounds have been extensively studied and reviewed for their potential for genotoxicity and carcinogenicity, with the consistent conclusion being that they lack genotoxic and carcinogenic potential [12,13,20,21,96,97]. Steviol glycosides have established ADIs of 0–12 mg/kg/d (United States FDA) and 0–4 mg/kg/day (JECFA and EFSA; expressed as steviol equivalents) (Table 1).

#### Genotoxicity and mechanistic data

Genotoxicity testing of steviol glycosides has largely been negative, showing no consistent evidence of genotoxicity, including in tests performed to detect mutagenicity, chromosomal aberrations, micronuclei formation, and general DNA damage [13]. Although a few in vitro studies have reported some positive results, EFSA concluded in its evaluation that these studies were unreliable, noting a number of limitations, including that OECD guidelines were not followed, critical methodological details were lacking, and/or the findings were the result of secondary toxicity from high doses used. In addition, EFSA noted that some in vitro studies reported positive results for metabolites of steviol glycosides that are not considered relevant to humans. EFSA further noted that in vivo studies conducted in rodents after oral administration showed no evidence of genotoxicity [13]. Genotoxicity tests conducted since the EFSA evaluation have been negative. In an HTS study designed to detect genotoxicity induced by small molecules, steviol glycosides were shown to be inactive in all 17 genotoxicity assays of the NTP ToxCast/Tox21 program [23]. In addition, in a recent systematic analysis of mechanistic data across over 900 endpoints relevant to the KCs of carcinogens paradigm, including those related to genotoxicity, steviol glycosides demonstrated an overall lack of KC-related activity, with the authors concluding that steviol glycosides lack both genotoxic and carcinogenic potential [97]. For several KCs (e.g., oxidative stress, inflammation, cell proliferation), it was observed that steviol glycosides had beneficial activity (anti-inflammatory, antioxidant, and antiproliferative).

#### Animal carcinogenicity

The lack of genotoxic potential is consistent with the lack of tumor response in 2-y rodent cancer bioassays conducted on steviol glycosides [20,21,97]. Three GLP-compliant standard rodent cancer bioassays have been conducted on steviol glycosides and have demonstrated no treatment-related increases in the formation of tumors or non-neoplastic lesions; a decreased incidence of mammary adenomas in female rats treated with Stevioside compared with controls was reported in 1 study [98]. An additional study conducted in transgenic mice genetically engineered to be particularly susceptible to pancreatic cancer showed no increased incidence of tumors [43].

#### Weight-of-evidence conclusion

Taken together, the available experimental evidence supports previous conclusions that steviol glycosides are not genotoxic or carcinogenic.

### Sucralose

#### Regulatory status and ADI

The genotoxic potential and carcinogenicity of sucralose have been extensively investigated over >3 decades in a number of in vitro and animal toxicity studies, both as part of its initial approval as a food additive by United States FDA in 1998 (ADI of 0–5 mg/kg/d) and in the 25 y since, with the overall evidence supporting the conclusion that sucralose is not genotoxic or carcinogenic (Table 1). Information on the genotoxicity and carcinogenicity of sucralose largely comes from evaluations of relevant in vitro and in vivo data by United States FDA in 1998 and by SCF in 2000 (ADI of 0–15 mg/kg/d), as part of their authoritative decisions [14,15], along with additional studies and systematic reviews published since then.

#### Genotoxicity and mechanistic data

The United States FDA and SCF both concluded that sucralose lacks genotoxic and carcinogenic potential, with the data considered negative or inconclusive for mutagenicity and genotoxicity, and negative for carcinogenicity. Although United States FDA noted inconclusive results for some genotoxicity tests, the agency concluded that any inconclusive genotoxicity results are superseded by the results of animal carcinogenicity studies (i.e., 2-y rodent bioassays) demonstrating that sucralose is not carcinogenic, as these assays "are more direct and complete tests of carcinogenic potential" [14].

Additional genotoxicity studies published since the United States FDA and SCF evaluations have demonstrated overall that sucralose is nonmutagenic and lacks genotoxic potential [26,99,100]. Although some inconclusive and/or positive results have been reported from tests conducted in vitro using cultured cells or in fish [27,101,102], these studies have been noted to have a number of shortcomings that limit the applicability of their results, including the misinterpretation of assay findings and the use of nonstandard methodologies [20,21]. In addition, in vivo genotoxicity tests evaluating the same and additional endpoints have been negative [99,100]. Further, a recent systematic analysis of mechanistic data across the 10 KCs of carcinogens, including genotoxicity, with integration of evidence from animal carcinogenicity studies, demonstrated an overall lack of activity associated with sucralose, with the authors concluding that sucralose is unlikely to be carcinogenic in humans [103]. Subsequent systematic reviews further concluded that there is no evidence to justify a modification of the United States FDA and SCF conclusions that sucralose lacks genotoxic and carcinogenic potential [20,21].

Since then, in 2023, a controversial study by Schiffman et al. [104] claimed to have identified sucralose-6-acetate (S6A) as a novel in vivo genotoxic acetylated metabolite of sucralose from a re-analysis of data obtained in rats [105]. This conclusion was based on an unpublished visual comparison of chromatograms produced years apart, although the data are not shown and do not appear to be publicly available. Under normal physiological conditions, sucralose is considered exceedingly stable, with very limited capability for biotransformation; this is particularly true at the hydroxyl groups where acetylation would have to occur to form S6A [106]. Consistent with this limited reactivity, no evidence for the in vivo acetylation of sucralose has been observed in previous pharmacokinetic and metabolism studies of sucralose [107,108]. Overall, the recent Schiffman et al. [104] study appears to be of low quality and limited reliability, with a number of critical limitations affecting the interpretation of results. If S6A were an in vivo metabolite of sucralose, and capable of producing genotoxicity and/or carcinogenicity, these effects would have been observed in the many in vivo genotoxicity and carcinogenicity studies previously conducted on sucralose. Ames testing, one of the most widely used in vitro assays in toxicology for assessing the mutagenic potential of chemical compounds (forming an essential component of the regulatory testing battery, including that recommended for food additives such as sucralose [109]), demonstrated that sucralose and the purported S6A metabolite are not mutagenic, but Schiffman et al. [104] reported that S6A was genotoxic in 2 in vitro tests [the MultiFlow DNA damage assay and the mammalian micronucleus (MN) assay]. In both of these assays, however, high levels of cytotoxicity may have confounded the results, as the doses at which S6A genotoxicity were observed exceeded assay recommendations [[110], [111], [112], [113]]. These doses are also unlikely to be relevant to humans, as they far exceed those expected to be achievable from human sucralose consumption. [Taking into consideration the (unsupported) claim by Schiffman et al. [104] that S6A is produced at a 10% rate in vivo relative to sucralose, and assuming 250 mg sucralose = 400 ng/mL blood concentration [114], it can be estimated that tens of thousands of cans of sucralose-sweetened beverages would have to be consumed over a short period of time (e.g., 2 hours) to reach S6A blood concentrations in the mM range at which the authors reported genotoxicity.] In addition, for the MultiFlow DNA damage assay, the source of some of the cutoff values used to determine genotoxicity is unclear; the source referenced by the authors [115] does not contain the cutoffs used to determine genotoxicity with metabolic activation. For the MN assay, the authors stated that significant results were only observed when all cell populations were scored for micronuclei, which is inconsistent with MN assay guidelines [[116], [117], [118]]. They also claimed to have observed a dose–response relationship in the MN assay results, but lower doses in the range that did not support this relationship were excluded. Schiffman et al. [104] do not discuss these limitations.

#### Animal carcinogenicity

The lack of genotoxicity for sucralose is supported by the conclusions reached by United States FDA and SCF regarding the lack of carcinogenic risk associated with sucralose, which were based on 2 standard GLP- and OECD-compliant 2-y rodent cancer bioassays conducted in male and female rats and mice that reported no treatment-related increased tumor incidence [119,120]. Since then, a third cancer bioassay, conducted at the Ramazzini Institute, has been described, with the authors reporting a significant dose-related increase in the number of malignant tumors in male (but not female) mice exposed to sucralose at the highest dose (16,000 ppm), with hematopoietic neoplasias making up the majority of the findings [121]. However, similar to the studies conducted for aspartame at the Ramazzini Institute (see above), this Ramazzini Institute study has been heavily criticized and the results considered unreliable [122]. A number of critical flaws related to the study design and reporting of the Soffritti et al. [121] results have been identified, including the use of a nonstandard dosing duration (dosing until death), the lack of historical control data, high levels of inflammation in the control groups, and the lack of a dose–response relationship [20,45,103,106,[122], [123], [124]].

#### Weight-of-evidence conclusion

Taken together, the body of experimental evidence supports the original conclusions by United States FDA and SCF that sucralose is neither genotoxic nor carcinogenic.

## Discussion

The overall weight of experimental evidence does not support the biological plausibility that the NSSs Ace-K, advantame, aspartame, cyclamate, neotame, saccharin, steviol glycosides, and sucralose could cause cancer in humans. This conclusion is consistent with the results of high-quality animal bioassays demonstrating a lack of tumorigenic response, and the results of numerous in vitro and in vivo studies investigating mechanisms that may be associated with cancer (e.g., genotoxicity) that have shown no or limited activity. Overall, these studies evaluated the NSSs over long periods of time at doses and levels that far exceed those associated with human consumption.

Although some genotoxicity assays have reported positive results for several of the NSSs, these assays have largely been from historical studies that did not conform to OECD guidelines or have been noted by authoritative bodies to be of low quality and limited relevance. In general, the interpretation of genotoxicity assay results is considered complex, as a large number of variables are known to confound the results. For determining whether a given agent causes cancer, animal cancer bioassays are considered the scientific standard, superseding those of genotoxic (and mechanistic) studies, as they are a more direct measure of carcinogenic potential.

Although saccharin has been shown to be carcinogenic to the rat bladder, the mechanism by which urinary crystals are formed in the rat bladder from saccharin exposure, resulting in later tumor formation, is not considered to be biologically plausible or relevant in humans. For other NSSs, animal bioassays have all been negative, with the exception of positive results reported for aspartame and sucralose from lifetime animal bioassays conducted at the Ramazzini Institute; however, these studies have subsequently been considered unreliable by authoritative bodies and the scientific community.

Overall, studies conducted at the Ramazzini Institute have been noted to suffer from a large number and variety of serious flaws, including with respect to quality control, study design and conduct, statistical analyses, and animal health [5,6,20,44,45,[49], [50], [51], [52], [53], [54], [55]]. In 2010, United States EPA concluded it would no longer use Ramazzini Institute data, particularly for hematopoietic cancers, due to issues with animal infections and tumor misdiagnosis [125]. For the aspartame and sucralose studies, there is no indication that microbiological monitoring for infections and disease was performed [44,54]. In addition, in a recent analysis that sought to compile a historical control database from published Ramazzini Institute studies, high variability in tumor incidence across the control groups of the aspartame and sucralose studies was observed, and all tumor incidences reported in the treatment groups fell below or within the range of historical controls [45].

In recent years, carcinogenicity evaluations by IARC and other authoritative bodies have become increasingly focused on mechanistic evidence related to the KCs of carcinogens paradigm. Currently, evidence of KC activity can form the basis for classifying agents as *possibly carcinogenic to humans* (Group 2B), when the human and animal evidence are inadequate and less than sufficient, respectively. Evidence of KC activity can also upgrade a classification from Group 2B to 2A (*probably carcinogenic to humans*) when there is limited human evidence and less than sufficient animal evidence [126].

Although the evidence overall has shown that NSSs lack KC-related mechanistic activity, for aspartame, IARC used limited mechanistic activity for KCs, including oxidative stress (KC #5), to lend support to its controversial decision to classify aspartame as Group 2B [4]. However, KC evidence lacks specificity and is not informative for determining the actual likelihood of an agent causing cancer in humans [[127], [128], [129], [130]]. This is because the KC paradigm is based on empirical observations of certain chemical and biological properties, rather than direct evidence of carcinogenicity (e.g., from animal cancer bioassays), and activity for a given KC does not necessarily mean an agent can (or will) cause cancer through this activity [131]. For example, as noted by IARC, compounds that do not cause cancer can induce oxidative stress [126]. Similarly, not all genotoxins are carcinogens [130]. Although KC information may be useful for the identification of mechanisms of carcinogenesis once an agent has been established to cause cancer, when used in isolation, this information may lead to overly simplistic and misleading conclusions. These limitations should continue to be considered, as the increasing use of KC information by IARC and other agencies is anticipated.

The lack of carcinogenicity for NSSs in animal bioassays is consistent with the results of epidemiology studies, which have shown no consistent associations between NSS intake and cancer risk. In conjunction with this evaluation, we also conducted a large systematic review of 90 available epidemiology studies of Ace-K, aspartame, cyclamate, saccharin, sucralose, or nonspecific NSSs in aggregate [e.g., diet sodas, artificially sweetened beverages] and cancer, published through Fall 2024 [16]. There were no consistent associations between the consumption of any NSS (or NSSs in aggregate) and any type of cancer. In addition, there was no evidence for any dose–response relationship for any type of NSS (or NSSs in aggregate) and any type of cancer.

The aim of this review was to provide a comprehensive evaluation and synthesis of the current status of the animal and mechanistic evidence together for the 8 common NSSs within the context of ingredient safety, relying on high-quality agency and regulatory analyses and documents to identify additional data, and providing insights into recent controversies and developments in the field. Integrating the available animal, mechanistic, and human evidence together supports the conclusion that these NSSs do not pose a genotoxic or carcinogenic risk to humans.

In conclusion, evaluation of the available animal and mechanistic evidence for the NSSs Ace-K, advantame, aspartame, cyclamate, neotame, saccharin, steviol glycosides, and sucralose indicates these compounds lack genotoxic and carcinogenic potential. Overall, these studies show no human-relevant evidence for carcinogenicity in animal models and no consistent or compelling evidence for any biologically plausible mechanism or MoA by which any of these NSSs could cause cancer in humans.

## Author contributions

The authors’ responsibilities were as follows – SAM, JEG: conducted research and analyzed data; JEG: had primary responsibility for final content; and all authors: designed research, wrote the paper, read and approved the final manuscript.

## Data availability

Data described in the manuscript, codebook, and analytic code will be made available on request pending application and approval.

## Funding

ABA provided funding for this paper, which was written during the authors’ normal course of employment.

## Conflict of interest

All authors are employed by Gradient or the American Beverage Association (ABA). Gradient is an environmental and risk sciences consulting firm. ABA is the trade association that represents America’s nonalcoholic beverage industry. This paper represents the professional opinions of the authors and not those of ABA.
